# Transcription and FACT facilitate the restoration of replication-coupled chromatin assembly defects

**DOI:** 10.1038/s41598-023-38280-w

**Published:** 2023-07-14

**Authors:** Marta Barrientos-Moreno, Douglas Maya-Miles, Marina Murillo-Pineda, Sara Fontalva, Mónica Pérez-Alegre, Eloísa Andujar, Félix Prado

**Affiliations:** 1 Department of Genome Biology, Andalusian Molecular Biology and Regenerative Medicine (CABIMER), CSIC‑University of Seville‑University Pablo de Olavide, Seville, Spain; 2 Genomic Unit, Andalusian Molecular Biology and Regenerative Medicine Center (CABIMER), CSIC‑University of Seville‑University Pablo de Olavide, Seville, Spain

**Keywords:** Biological techniques, Cell biology, Genetics, Molecular biology

## Abstract

Genome duplication occurs through the coordinated action of DNA replication and nucleosome assembly at replication forks. Defective nucleosome assembly causes DNA lesions by fork breakage that need to be repaired. In addition, it causes a loss of chromatin integrity. These chromatin alterations can be restored, even though the mechanisms are unknown. Here, we show that the process of chromatin restoration can deal with highly severe chromatin defects induced by the absence of the chaperones CAF1 and Rtt106 or a strong reduction in the pool of available histones, and that this process can be followed by analyzing the topoisomer distribution of the 2µ plasmid. Using this assay, we demonstrate that chromatin restoration is slow and independent of checkpoint activation, whereas it requires the action of transcription and the FACT complex. Therefore, cells are able to “repair” not only DNA lesions but also chromatin alterations associated with defective nucleosome assembly.

## Introduction

The structural unit of chromatin is the nucleosome, formed by 147-bp of DNA wrapped around an octamer of core histones. Nucleosomes are not randomly distributed on the genome but occupying preferential positions with respect to the DNA sequence. These positions are established by the DNA sequence composition and the action of chromatin remodelers, general regulatory factors (GRFs) and transcription^1,2^. Nucleosome position is critical for genome regulation, as it dictates DNA accessibility and therefore the processivity of essential processes like transcription, replication, DNA repair and homologous recombination. Accordingly, chromatin alterations may cause from a severe loss of cell fitness to lethality, and are associated with cancer, neurodevelopmental disorders and aging^3–6^. More specifically, chromatin assembly defects can lead to replication fork instability, generating DNA lesions that need to be repaired^7–9^.

Nucleosomes are assembled during S phase through a process that is coupled to DNA synthesis, with the first nucleosome deposited ~ 250-bp behind the replication fork^10^. Replication-coupled (RC) nucleosome assembly involves the action of histone chaperones that interact with replisome components to incorporate both parental and newly synthesized histones into the nascent strands^11,12^. In yeast, the chaperone Asf1 presents newly synthesized H3/H4 dimers to the acetyltransferase Rtt109 for its acetylation at H3K56, a modification that enhances the affinity of the dimer for the histone chaperones CAF1 (formed by Cac1, Cac2 and Cac3) and Rtt106^13–16^. These two chaperones play redundant roles in the deposition of new histones, and only the lack of both complexes strongly affects this process^16^. However, a double mutant lacking CAF1 and Rtt106 is not significantly affected in cell growth, which is explained by the action of the FACT (facilitates chromatin transcription) complex (formed in yeast by the histone chaperones Spt16 and Pob3)^17,18^.

Paradoxically, building up chromatin at nascent strands during DNA replication is associated with the disruption of parental chromatin ahead of the fork^19^. These chromatin-disruptive activities are required for the advance of the replisome and facilitated by the process of parental histone recycling^20,21^. It was early observed that the newly assembled differed from the bulk chromatin^22–24^, reflecting the need to recover the original chromatin features in order to maintain genome functionality. This process, termed chromatin maturation, involves both nucleosome repositioning and recovery of parental histone marks diluted by the incorporation of new histones, and has been studied in the last years in yeast, *Drosophila* and mouse embryonic stem cells (mESCs) using genome-wide approaches that compare nascent and mature chromatin^25–33^. These studies have mostly focused on the chromatin organization of the gene units, as both transcription factors (*cis* and *trans*) and transcription activity are major determinants of nucleosome positioning^1,2,34^. A conserved feature across eukaryotes is the presence of a nucleosome-free region (NFR) at promoters flanked by two well positioned nucleosomes (− 1 and + 1), and a regularly spaced nucleosome array downstream the + 1 nucleosome with fuzzier positions as nucleosomes are more distant from the promoter^2,35^. Chromatin organization at transcription units is lost at nascent chromatin in all analyzed cells, with gaining of nucleosomes at the NFRs and global loss of nucleosome positioning. This suggests that RC-chromatin assembly outcompetes DNA binding proteins, including transcription factors, offering an opportunity to modify chromatin patterns^26,29,30^. The recovery of matured nucleosome patterns at promoters and enhancers in *Drosophila* and mESCs takes from 30 to 120 min, and at least in mESCs it requires active transcription except for enhancers with nucleosome destabilizing DNA sequences^26,29^. In yeast, this process is faster (less than 5 min), does not require transcription (likely due to the presence of AT-rich DNA sequences at its promoters), and is associated with the binding of GRFs^25,27,28,30–32^. In contrast to promoters, chromatin maturation at the gene bodies is slower and requires transcription elongation both in yeast and mESCs^27,29,32^.

Unexpectedly, yeast cells are viable under conditions that cause severe chromatin integrity defects, as the absence of both CAF1 and Rtt016 or a strong reduction in the pool of available histones^16,36^. This likely reflects the capacity of cells to buffer huge oscillations in gene expression and deal with high levels of genetic instability. However, some of the studies on chromatin maturation have shown that cells are able to partially restore chromatin alterations induced by the absence of CAF1, Asf1 or Rtt109^26,28,37^. Here, we have used a plasmid-based topological assay and genome-wide nucleosome profiling to show that cells are able to restore the highly severe loss of chromatin integrity induced in *cac1∆ rtt106∆* and histone-depleted mutant cells. Furthermore, we show that chromatin restoration is facilitated by the action of transcription and the FACT complex.

## Results

### Defective RC-nucleosome deposition causes transient changes in DNA topology and chromatin structure of the 2µ plasmid

**Figure 1:**
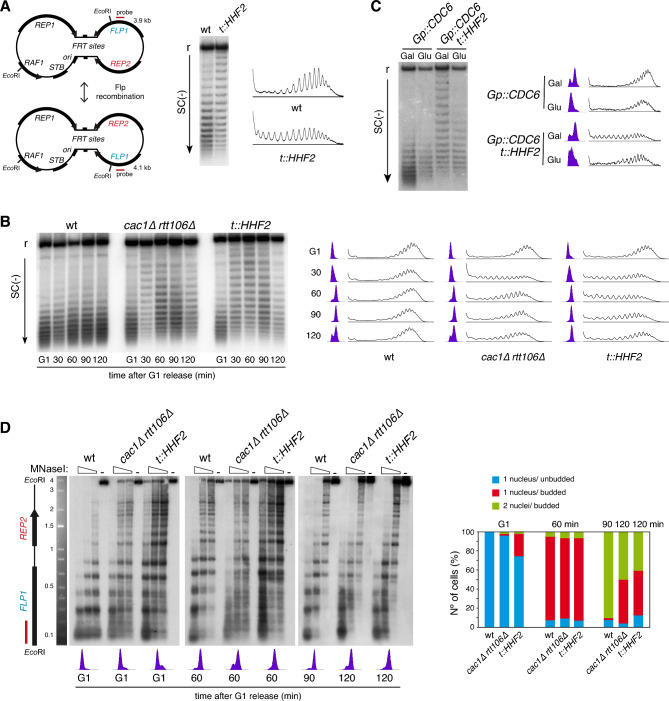
Defective replication-coupled histone deposition causes transient changes in DNA topology and chromatin structure of the 2µ plasmid. (**A**) Plasmid topoisomer distribution of the 2µ plasmid in asynchronous cultures of wild type and *t::HHF2* cells. A scheme of the two versions of the 2µ plasmid generated by Flp recombination, with the two unique halves and the intervening inverted repeat (*FRT*), is shown on the left. (**B**) Plasmid topoisomer distribution of the 2µ plasmid in wild type, *cac1∆ rtt106∆* and *t::HHF2* cells synchronized in G1 and released into fresh medium for different times. (**C**) Plasmid topoisomer distribution of the 2µ plasmid in *Gp::CDC6* and *Gp::CDC6 t::HHF2* cells synchronized in G1 and released into fresh medium in galactose or glucose-containing medium to express or not Cdc6, respectively. (**A**–**C**) Cell cycle progression and topoisomers profiles are shown. r and SC(−) indicate relaxed and negative supercoiling, respectively. Images show only the distribution of monomeric forms, as the higher-order forms reflect multimeric structures associated with the rolling circle replication mechanism of the 2µ plasmid ^45^, which are not a good readout to detect chromatin alterations (see supplementary Figures for complete and cropped images). (**D**) Chromatin structure of the *Eco*RI fragment spanning the *FLP1* (bottom) and *REP2* (top) genes from the 2µ plasmid in wild type, *cac1∆ rtt106∆* and *t::HHF2* cells synchronized in G1 and released into fresh medium for different times. See Fig. 1A for the position of the probe at the *Eco*RI fragment. Samples were run into different gels due to space limitations, and processed in parallel. Cell cycle stage of wild type, *cac1∆ rtt106∆* and *t::HHF2* cells was determined by FACS, cell morphology and DAPI (4′,6′-diamidino-2-phenylindole) staining of nuclei. (**A**–**D**) Original gels are presented in Fig. S1. The experiments were repeated at least twice with similar results.

Partial depletion of histones causes a dramatic loss of chromatin integrity that is associated with a loss of negative supercoiling^38^. This loss of negative supercoiling is due to the fact that the assembly of each nucleosome introduces one negative superhelical turn^39^. This topological change can be detected by analyzing the distribution of topoisomers of a plasmid in chloroquine-containing gels, and has been extensively used to address chromatin alterations in vivo and in vitro^36,38,40–44^. Specifically, the loss of negative supercoiling in histone-depleted yeast cells can be detected by analyzing the endogenous 2µ plasmid in a strain in which the only source of histone H4 is under control of the doxycycline-regulated *tet* promoter (*t::HHF2* strain; Fig. 1A)^36^. The topological behavior in response to histone depletion of the 2µ plasmid is similar to that displayed by a centromeric plasmid, but it is more sensitive because its multicopy nature^38^. The 2µ plasmid is organized as two unique regions separated by inverted repeats (*FRT* sites). These repeats can recombine leading to equal amounts of two plasmids that differ in the orientation of one unique region with respect to the other (Fig. 1A, left panel). Although the plasmid is replicated through a canonical semiconservative mechanism from the origin, this recombination system helps to maintain the copy number by a DNA amplification mechanism that leads to rolling circle replication intermediates^45^. To focus on the nucleosome-associated topological changes, only the distribution of the monomeric forms is analyzed^36,38,44^.

The aforementioned DNA supercoiling analyses were carried out in asynchronous cultures. To understand the cell cycle dynamics of these topological changes, cells were synchronized in G1 and released into fresh medium under conditions of *HHF2* repression (0.25 µg/ml dox) (Fig. 1B). Whereas the distribution of topoisomers was similar along the cell cycle in the wild type strain, the *t::HHF2* mutant displayed wild-type topological levels in G1 and a strong but transient loss of negative superhelical density from early S phase to G2/M (Fig. 1B).

To confirm that this transient defect in DNA topology was associated with the process of RC-nucleosome assembly, *t::HHF2* cells were synchronized in G1 and released into S phase in the absence of Cdc6, which is essential for replication initiation but not for further cell-cycle events^46^. As shown in Fig. 1C, DNA replication was required for the transient loss of negative supercoiling in histone-depleted cells. To further demonstrate that the effect of histone depletion on DNA topology is a consequence of a defect in the process of RC-chromatin assembly, we analyzed the distribution of topoisomers during the cell cycle in a *cac1∆ rtt106∆* mutant. This mutant also displayed wild-type topological levels in G1 and a transient loss of negative supercoiling during S-G2/M (Fig. 1B). The main difference was that the recovery of negative supercoiling was faster in *cac1∆ rtt106∆* than in *t::HHF2* cells.

These results suggest that the alterations in chromatin structure induced by defective histone deposition occur transiently during S phase and are post-replicatively restored. To confirm this, we analyzed the pattern of nucleosomes in the 2µ plasmid by indirect-end labelling of MNaseI-treated cells at different times during the cell cycle. We focused on the chromatin structure of an *Eco*R1 fragment containing the *FLP1* and *REP2* genes (Fig. 1A, left panel). The *t::HHF2* and *cac1∆ rtt106∆* mutants displayed a much more altered chromatin structure 60 min after G1 release than in G1, and these alterations were partially restored 60 min later (Fig. 3D). These time points correspond to G2/early mitosis and late mitosis, respectively, as determined by FACS, cell morphology and nuclei staining (Fig. 1D; note that the *t::HHF2* and *cac1∆ rtt106∆* mutants accumulate at metaphase due to checkpoint activation^7,47^). It is worth noting that *t::HHF2* and *cac1∆ rtt106∆* shared similar chromatin alterations; high accessibility of the nucleosomal DNA and similar modified bands.

### Chromatin assembly defects in *cac1∆ rtt106∆* are largely restored genome wide

**Figure 2:**
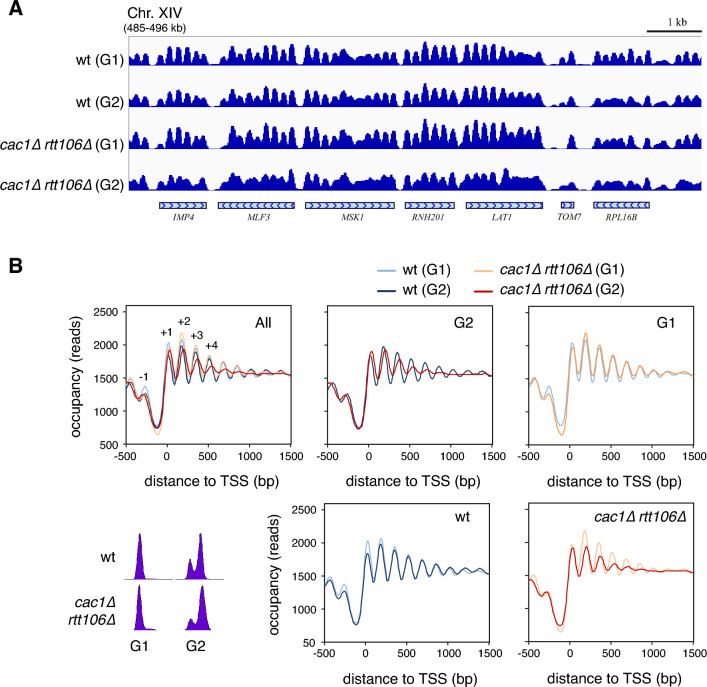
Chromatin assembly defects in *cac1∆ rtt106∆* are largely restored genome wide. (**A**–**B**) Genome-wide nucleosome profiles by MNase-seq of wild type and *cac1∆ rtt106∆* cells synchronized in G1 and released into fresh medium for 60 min until G2. A representative nucleosome profile (**A**) and the occupancy profiles for all yeast genes aligned relative to the TSS (**B**) are shown. Cell cycle progression was followed by flow cytometry. The analysis was performed with two biological replicates.

We asked if the loss and further recovery of chromatin integrity of the 2µ plasmid in nucleosome-deposition mutants reflected a genome-wide process. For this, we performed high-throughput sequencing of MNase I–digested chromatin (MNase-seq) followed by dynamic analysis of nucleosome position and occupancy by sequencing (DANPOS)^48^, which allows nucleosomes to be mapped along the whole genome. We analyzed the nucleosomal landscape of *cac1∆ rtt106∆* and wild type cells both in G1 and G2 phases (60 min after G1 release) to allow completion of genome replication. The absence of CAF1 and Rtt106 during DNA replication caused severe defects in the distribution of nucleosomes in G2 (Fig. 2A). This loss of chromatin integrity became particularly evident by a strong reduction in the amplitude of the nucleosomal oscillation in G2, which indicates a loss of nucleosome phasing (Fig. 2B, G2 panel). This chromatin defect was less severe in S than in G2 phase (compare panel G2 in Fig. 2B with Fig. S2A), consistent with an accumulation of affected genes as replication is completed.

Nucleosome positioning became slightly better defined in G1 in the wild type strain, especially around the NFR where nucleosomes − 1 to + 3 increased their occupancy (Fig. 2B, wt panel). The similarity in the nucleosomal profiles of G2 and G1 was due to the fast maturation of the newly assembled chromatin during S phase^25,27,28,31^. In contrast, the *cac1∆ rtt106∆* mutant showed a significant drop in the occupancy of nucleosome − 1 in G1 relative to the wild type strain (Fig. 2B, G1 panel). Indeed, the analysis of individual genes revealed a loss not only of this nucleosome but also from gene body nucleosomes in multiple genes in G1 (Fig. S2B). This phenotype is likely related to the replication-independent, transcription-dependent role of Rtt106 preventing spurious transcription and maintaining promoter fidelity by histone replacement^49,50^. Apart from this specific alteration, chromatin integrity was largely restored in the *cac1∆ rtt106∆* mutant in G1 (Fig. 2A and B; compare mutant and wild type profiles in G2 and G1 panels). In conclusion, cells are able to correct severe chromatin alterations occurring during the process of RC-nucleosome assembly, and these changes are associated with a transient loss of plasmid negative supercoiling. Therefore, we used this plasmid topology assay to study the chromatin restoration process.

### Chromatin restoration in RC-nucleosome deposition mutants is independent of cell cycle arrest

**Figure 3:**
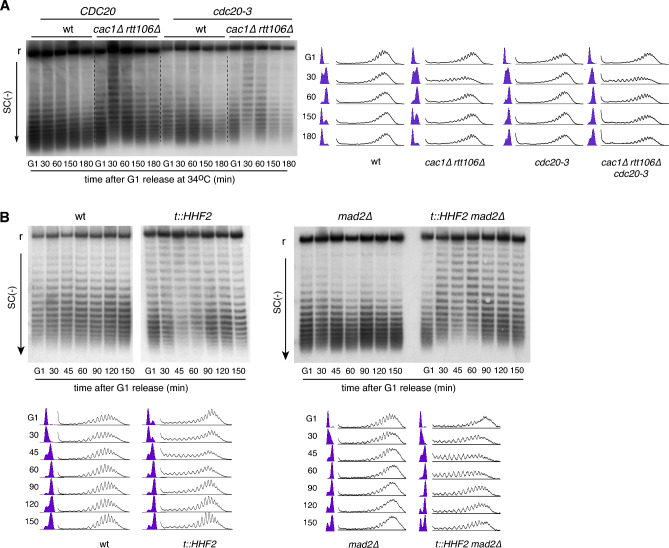
Chromatin restoration in CA-nucleosome deposition mutants is independent of cell cycle arrest. (**A**) Plasmid topoisomer distribution of the 2µ plasmid in wild type, *cdc20-3*, *cac1∆ rtt106∆* and *cac1∆ rtt106 cdc20-3* cells at 34 °C. Cells were synchronized in G1, released into fresh medium for 60 min and resynchronized in G1. (**B**) Plasmid topoisomer distribution of the 2µ plasmid in wild type, *t::HHF2*, *mad2∆* and *t::HHF2 mad2∆* cells synchronized in G1 and released into fresh medium for different times. Samples were run into different gels due to space limitations, and processed in parallel. Cell cycle progression and topoisomer profiles are shown. r and SC(−) indicate relaxed and negative supercoiling, respectively. Cropped images show only relaxed and negatively supercoiled topoisomers. Original gels are presented in Fig. S3. The experiments were repeated at least twice with similar results.

The shift in the distribution of topoisomers induced by defective histone supply in *t::HHF2* and *cac1∆ rtt106∆* cells was largely restored to wild-type levels in mitosis (Fig. 1B). To confirm that chromatin restoration occurred before the metaphase-anaphase transition, we repeated the plasmid supercoiling analysis in *cac1∆ rtt106∆* cells expressing *cdc20-3*, a thermosensitive allele of the APC cofactor Cdc20 that causes a metaphase arrest at restrictive temperature^51^. In this case, G1-released S phase cells were washed and resuspended into fresh medium with α-factor for G1 resynchronization. The recovery of plasmid negative supercoiling occurred with similar kinetics with and without cell cycle-induced arrest (Figs. 3A and S3A), indicating that chromatin restoration of the 2µ plasmid occurs before anaphase.

Chromatin assembly mutants transiently arrest in metaphase^7,14,47,52,53^. Therefore, we wondered if this arrest was required for the recovery of plasmid negative supercoiling. Most chromatin assembly mutants, including the double mutant *cac1∆ rtt106∆*, arrest in metaphase due to the activation of the DNA damage checkpoint (DDC)^7,14,52,53^. We observed that a triple mutant *cac1∆ rtt106∆ mec1∆*, defective in DDC activation, was proficient in the recovery of negative supercoiling (Fig. S3B). However, chromatin assembly defects can also lead to a metaphase arrest by activation of the spindle-assembly checkpoint (SAC), as it is the case of the *t::HHF2* mutant^47^. These cells, interestingly, do not activate the DDC despite the accumulation of DNA damage (Fig. S3C)^47^. Therefore, *t::HHF2 mad2∆*, lacking a functional SAC, is an optimal mutant to address if cell cycle arrest is required for chromatin restoration. The elimination of the metaphase arrest in a SAC-deficient *mad2∆* background did not alter the kinetics of plasmid supercoiling of the *t::HHF2* mutant (Fig. 3B). The recovery of negative supercoiling was slightly worse in *t::HHF2 mad2∆* than in *t::HHF2* cells; however, this difference is likely associated with the accumulation of dead cells in mitosis and G1 by chromosome mis-segregation^47^. Therefore, the post-replicative restoration of the chromatin assembly defects is independent of cell cycle arrest.

### Restoration of *cac1∆ rtt106∆*-mediated chromatin assembly defects are facilitated by transcription

**Figure 4:**
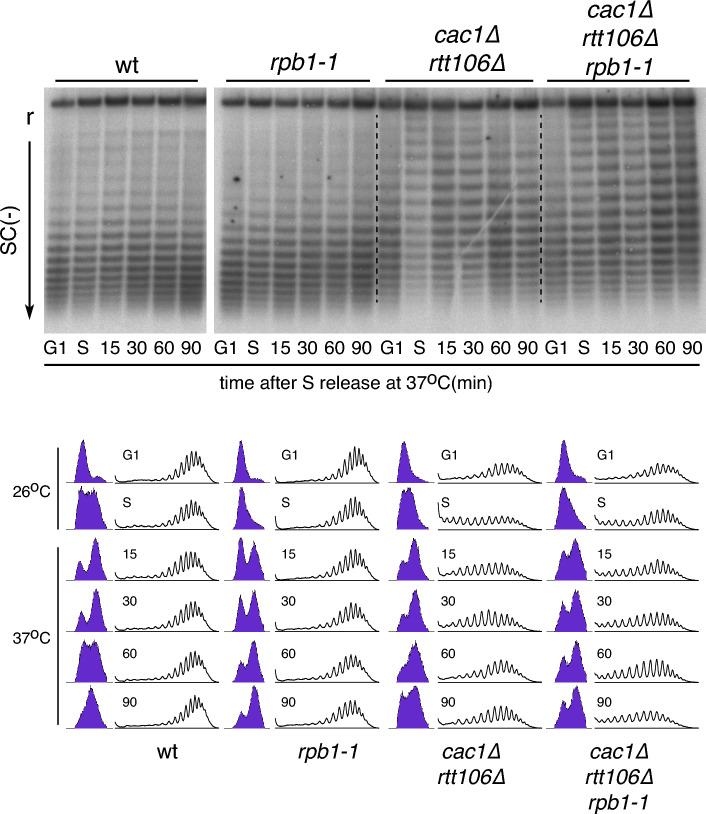
Restoration of *cac1∆ rtt106∆*-mediated chromatin assembly defects are dependent on transcription. Plasmid topoisomer distribution of the 2µ plasmid in wild type, *rpb1-1*, *cac1∆ rtt106∆* and *cac1∆ rtt106 rpb1-1* cells synchronized in G1 and released into fresh medium until mid-S phase (60 min for *cac1∆ rtt106 rpb1-1* and 30 min for the rest) at 26 °C, and then shifted to and incubated with pre-heated fresh medium at 37 °C for the indicated times. Samples were run into different gels due to space limitations, and processed in parallel. Cell cycle progression and topoisomer profiles are shown. r and SC(−) indicate relaxed and negative supercoiling, respectively. Cropped images show only relaxed and negatively supercoiled topoisomers. Original gels are presented in Fig. S4B. The experiment was repeated twice with similar results.

Transcription activity helps to correctly position nucleosomes^34^, and accordingly it is required for chromatin maturation^27,29,32^. Therefore, transcription provides a potential mechanism to restore post-replicatively a loss of chromatin integrity occurring during genome duplication. To address the relevance of transcription in the recovery of the *cac1∆ rtt106∆*-mediated chromatin assembly defects, we followed the distribution of plasmid topoisomers along the cell cycle in cells expressing a wild type or a thermosensitive allele of the largest subunit of RNAPII (*rpb1-1*)^54^. Since transcription was essential to exit from G1 (Fig. S4A), cells were shifted from permissive (26 °C) to restrictive temperature (37 °C) in the middle of S phase (peak of negative supercoiling loss; 30 min for all strains except for the triple mutant *cac1∆ rtt106∆ rpb1-1* that required 60 min because of a slower G1 exit). After the shift, cells were maintained at restrictive temperature for 90 min. The absence of transcription post-replication did not affect the pattern of plasmid supercoiling during the cell cycle (Fig. 4; compare *rpb1-1* with wt). The loss of negative supercoiling in the triple mutant *cac1∆ rtt106∆ rpb1-1* was less pronounced than in the double mutant *cac1∆ rtt106∆* (Fig. 4; compare the shift in topoisomers from G1 to S phase in both strains), suggesting that the *rpb1-1* allele slightly affected the accumulation of chromatin assembly defects at permissive temperature. Importantly, the absence of transcription strongly reduced the recovery of plasmid negative supercoiling in the *cac1∆ rtt106∆* mutant (Fig. 4; compare *cac1∆ rtt106∆ rpb1-1 *and *cac1∆ rtt106∆* strains at 60–90 min after the shift to restrictive temperature), even though a slight recovery was observed in the triple mutant at later times (Fig. 4; compare 60 and 90 min after the shift in the *cac1∆ rtt106∆ rpb1-1* mutant). Therefore, transcription helps to restore the loss of chromatin integrity associated with defective RC-nucleosome assembly.

### FACT helps to restore *cac1∆ rtt106∆*-mediated chromatin assembly defects

**Figure 5:**
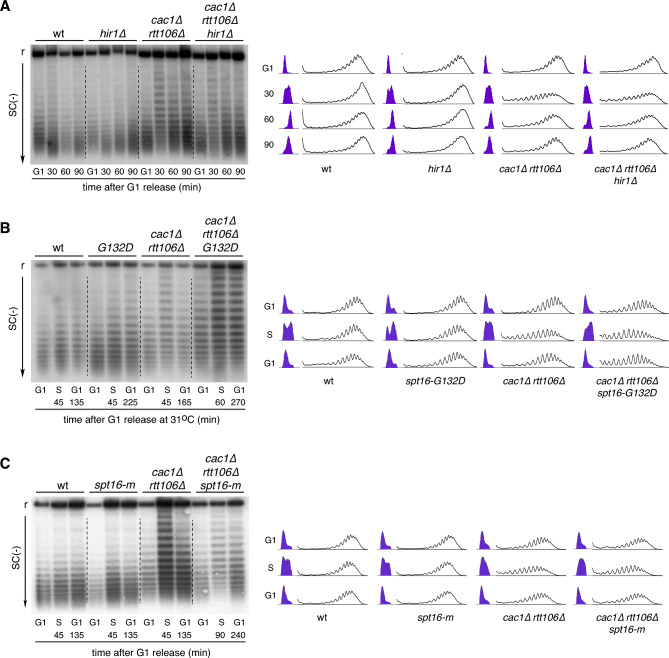
FACT helps to restore *cac1∆ rtt106∆*-mediated chromatin assembly defects. (**A**) Plasmid topoisomer distribution of the 2µ plasmid in wild type, *hir1∆*, *cac1∆ rtt106∆* and *cac1∆ rtt106 hir1∆* cells synchronized in G1 and released into fresh medium for different times. (**B**) Plasmid topoisomer distribution of the 2µ plasmid in wild type, *spt16-G132D*, *cac1∆ rtt106∆* and *cac1∆ rtt106∆ spt16-G132D* cells synchronized in G1 at 26 °C and released into fresh medium at 31 °C; in S phase, cells were collected, washed to eliminate the pronase and released into pre-heated medium with α-factor till the following G1. (**C**) Plasmid topoisomer distribution of the 2µ plasmid in wild type, *spt16-m*, *cac1∆ rtt106∆* and *cac1∆ rtt106∆ spt16-m* cells synchronized in G1 and released into fresh medium; in S phase, cells were collected, washed to eliminate the pronase and released into pre-heated medium with α-factor till the following G1. The experiment was repeated twice with similar results. (**A**–**C**) Cell cycle progression and topoisomer profiles are shown. r and SC(−) indicate relaxed and negative supercoiling, respectively. Cropped images show only relaxed and negatively supercoiled topoisomers. Original gels are presented in Fig. S5. The experiments were repeated at least twice with similar results.

Two chromatin-remodeling pathways have been proposed to maintain nucleosome integrity during transcription. The first pathway depends on Asf1 and the HIR complex and plays a major role at the intergenic region by nucleosome exchange. FACT and Spt6 are the major effectors of the second pathway and are more–but not exclusively—dedicated to the reassembly of histones throughout the gene bodies^55^. First, we addressed the role of the HIR complex (formed by Hir1, Hi2 and Hir3 in *S. cerevisiae*), which has been involved both in chromatin maturation and restauration of *cac1∆*-associated chromatin defects^27,28^. The absence of the HIR complex in cells lacking its major subunit (Hir1) did not prevent the recovery of plasmid negative supercoiling in *cac1∆ rtt106∆* cells (Fig. 5A), suggesting that it is not required for the recovery of chromatin integrity in this mutant.

To address the relevance of the second pathway in restoring replication-coupled chromatin assembly defects, we analyzed the effect on DNA topology of the thermosensitive allele *spt16-G132D*; this mutation affects the stability of Spt16 at restrictive temperatures^56^. Since the elimination of Spt16 at restrictive temperature causes transcription-associated chromatin assembly defects^56^, we performed the analysis in cells synchronized in G1 at permissive temperature (26 °C) and released at semi-permissive temperature (31 °C) until the following G1 phase, which required different times for each strain. The logic behind is to allow a complete restoration of the *cac1∆ rtt106∆*-induced chromatin defects. At this semi-permissive temperature, plasmid topology was hardly affected in the *spt16-G132D* mutant (Fig. 5B). Importantly, the loss of negative supercoiling induced by the absence of CAF1 and Rtt106 was not recovered in the triple mutant *cac1∆ rtt106∆ spt16-G132D*. Indeed, we observed a slight but reproducible loss of negative supercoiling in the triple mutant in G1, suggesting that the Spt16-G132D protein is partially defective in chromatin restoration even at permissive temperature. In contrast to *spt16-G132D*, a *spt16-m* allele that specifically affects the RC-histone deposition activity of FACT^17^, was able to restore the chromatin assembly defects induced by the absence of CAF1 and Rtt106 (Fig. 5C). Therefore, the activity of FACT facilitates chromatin restoration after defective RC-nucleosome assembly.

## Discussion

The efficiency of the nucleosome deposition process during DNA replication eliminates chromatin characteristics, which are recovered through a maturation process that depends on DNA sequence composition, GRFs and chromatin remodeling factors, and transcription^25–33^. Several studies support that cells can restore chromatin alterations generated by mutations in nucleosome deposition factors in yeast (*caf1∆*, *asf1*∆ and *rtt109*) and *Drosophila* (Caf1-105 knockdown)^26,28,37^, although the mechanisms of restoration are poorly understood. In yeast, *asf1∆* and *rtt109∆* mutants delay histone deposition due to a lack of H3K56 acetylation that reduces histone delivery to CAF1 and Rtt106. Accordingly, chromatin is less affected in *asf1∆* and *rtt109∆* than in *cac1∆* and *rtt106*∆ mutants^25^. Yet, chromatin assembly defects in *cac1∆* and *rtt106∆* mutants are buffered by the action of Rtt106 and CAF1, respectively^7,16^. Here, we have shown by genome-wide MNase-seq that the severe loss of chromatin integrity induced during S phase by the absence of CAF1 and Rtt106 is largely restored post-replication, and that the process of chromatin restoration can be followed by analyzing the level of plasmid supercoiling along the cell cycle. This provides an alternative to the more expensive and time-consuming MNase-seq assay to screen for genetic requirements of the chromatin restoration process. Using this plasmid topology assay, we have shown that cells are able to restore even the severe chromatin defects induced by a strong reduction in the pool of available histones, and that chromatin restoration is facilitated by the action of transcription and the FACT complex. We have focused on the monomeric and not in the multimeric forms of the 2µ plasmid to study the connection between DNA topology and chromatin alterations, thus minimizing template-specific effects. In any case, chromatin dynamics is influenced by the structural and functional particularities of the analyzed regions, and therefore a deeper characterization will require genome-wide approaches.

Our genome-wide analysis shows that most chromatin assembly defects generated in *cac1∆ rtt106*∆ cells during DNA replication become restored in G1. However, chromatin was more altered in G2 than in S phase, which is consistent with the severe genome-wide chromatin assembly defects remaining in histone-depleted cells arrested in G2/M^57^. These results suggest that chromatin restoration is slower than chromatin maturation, which is completed in 5–20 min after replication fork passage^25,27,28^, yet highly efficient even under conditions that strongly disrupt the chromatin landscape as those induced in *cac1∆ rtt106*∆ and *t::HHF2* mutants. The accumulation of chromatin alterations and their “repair” during chromatin restoration can also be detected by following the distribution of plasmid topoisomers in RC-nucleosome assembly mutants during the cell cycle. These mutants display a strong and transient loss of plasmid negative supercoiling during the cell cycle as a consequence of RC-chromatin disruption. In contrast, the population of plasmid topoisomers does not change in the wild type, which reflects the speed and efficiency of the chromatin maturation process. Therefore, this assay allows to specifically follow chromatin restoration.

A common feature of chromatin assembly mutants is a metaphase arrest triggered by the activation of the DDC and/or SAC^7,14,47,52,53^. We show that this arrest is not required for chromatin restauration, although it likely provides time to coordinate this process with the repair of the DNA lesions that will activate the checkpoints.

Transcription activity is a major determinant of nucleosome position^34^, and it is required for chromatin maturation after DNA replication^27,29,32^. We show that transcription facilitates chromatin restauration. The genome-wide chromatin analysis showed that the loss of nucleosome phasing at G2 in the *cac1∆ rtt106*∆ mutant mainly affected the gene bodies, as previously observed in histone-depleted cells in G2/M^57^. The analysis of nascent chromatin at early time points in a *cac1∆* mutant showed nucleosome defects both at promoters (gain and loss of occupancy at the NFR and the flanking nucleosomes, respectively) and gene bodies (loss of phasing)^28^. Therefore, the promoter architecture of the *cac1∆ rtt106*∆ mutant is likely first reconstructed to prime active transcription and restore chromatin in the gene body as proposed for chromatin maturation in yeast, where the rapid binding of GRFs at promoters generate molecular landmarks that fix the positions of flanking nucleosomes^25,30^. This mechanism is also likely necessary during the restoration of a severely altered chromatin in order to provide a rule for the transcription machinery to properly reposition nucleosomes during elongation. However, it is unlikely that this process resembles chromatin maturation in the initial steps. During chromatin maturation, restructured promoters with bound GRFs are still refractory to RNAPII recruitment^31^, which explains why transcription is buffered for a while after replication^31,58,59^. In contrast, defective chromatin assembly in *asf1∆* and *cac1∆ rtt106∆* cells causes a transient accumulation of aberrant coding and non-coding transcripts behind the replication forks^37^. This effect is more pronounced in the *cac1∆ rtt106∆* mutant, likely because of its higher nucleosome deposition defects and the role of Rtt106 in preventing aberrant transcription^49,50^. We speculate that transcription from spurious initiation sites may slow the process of chromatin restoration because of the repositioning of nucleosomes without a correctly defined reference.

The requirement of transcription elongation for chromatin maturation supports a role for chromatin remodeling complexes traveling with RNAPII like CHD1 and ISW1b. In agreement with this possibility, nascent chromatin-associated alterations persist in the absence of these chromatin remodelers^25,27^. Less clear is the relationship with transcription for the HIR complex^27^, a chromatin remodeler that participates in replication-independent histone turnover, preferentially at intergenic regions^55,60,61^. The study of bulk nucleosome organization has also pointed to a role for the HIR complex in the restoration of *cac1∆*-induced nucleosome assembly defects^28^. Our plasmid topology assay did not reveal any role for the HIR complex in chromatin restoration in the *cac1∆ rtt106∆* mutant. Although the difference might be plasmid-specific, it cannot be excluded that the loss of nucleosome phasing in *cac1∆ hir1∆* cells reflects an additive effect of the absence of both complexes, as the *hir1∆* mutant by itself displayed a reduction in the amplitude of the nucleosomal oscillation on coding regions^28^.

FACT is a nucleosome remodeler complex with a critical role in nucleosome repositioning during transcription elongation. FACT travels with the RNAPII promoting the redeposition behind RNAPII of the original nucleosomes evicted during elongation through a stepwise mechanism of nucleosome disassembly-assembly that helps to maintain the epigenetic identity^55,62–66^. We have observed that the *spt16-G132D* mutant has no defects in the distribution of plasmid topoisomers but prevents the recovery of the negative supercoiling level lost during DNA replication in a *cac1∆ rtt106∆* mutant at semi-permissive temperature (31 °C). Therefore, the amount of Spt16 at this temperature seems to be sufficient to avoid a loss of nucleosomes during transcription but not to restore defective chromatin assembly. This suggests that the mechanism by which FACT restores chromatin is either different or requires more Spt16 than the mechanism by which FACT redeposits nucleosomes during transcriptional elongation. FACT is targeted to chromatin by recognizing the surface of disrupted nucleosomes generated mainly—but not exclusively –by transcription^56,67,68^. This observation, together with the ability of FACT to assemble nucleosomes led to Formosa and Winston to propose a role for FACT in the “repair” of disrupted nucleosomes^69^. It is likely that the dependency on transcription of the chromatin restoration process in *cac1∆ rtt106∆* cells reflects the need to disrupt nucleosomes to target FACT, which would be required at higher levels than in the wild type strain to additionally cope with displaced nucleosomes. An alternative but not exclusive possibility for the higher demand of Spt16 during chromatin restoration is that not only the position but also the integrity of some nucleosomes become affected in *cac1∆ rtt106∆* cells, targeting FACT in a transcription-independent manner.

In summary, cells are able to largely restore a severe loss of chromatin integrity induced under conditions of defective nucleosome assembly, providing a mechanism to buffer its impact on cell fitness. In addition, using plasmid topology as an easy and specific assay to study chromatin restoration, we have shown that this process requires the action of both transcription and FACT. This assay may help to uncover additional factors involved in chromatin restoration, as a previous step to a more detailed genome-wide characterization.

## Methods

### Yeast strains and growth conditions

Yeast strains used in this study are listed in Table S1. Cells were grown at 30 °C—unless otherwise indicated—in YPAD (experiments including *rpb1-1*, *spt16* and *Gp::CDC6* strains) or supplemented minimal medium (SMM) (rest). For metaphase synchronization, cells were treated with 15 µg/ml nocodazole for 1 h. For G1 synchronization, cells were grown to mid-log-phase and α factor was added twice at 90 min intervals at 0.5 μg/ml, except for *t::HHF2* strains (treated with 1 μg/ml) and *rpb1-1* strains (treated twice at 150 min intervals). Cells were then washed three times and released into fresh medium with 50 μg/ml pronase. For G1 resynchronization, cells released into S phase were washed and resuspended in fresh medium with α-factor at 1 μg/ml (*t::HHF2* strains) or 0.5 μg/ml (rest of strains) until G1. To induce nucleosome depletion, *t::HHF2* cells growing in the presence of 5 µg/ml doxycycline were shifted to 0.25 μg/ml during G1 synchronization and release. Cdc6 depletion was performed as previously described^70^. Briefly, *Gp::CDC6* cells were synchronized in metaphase in 2% galactose-containing medium with 1% DMSO and 15 µg/ml nocodazole for 2 h, shifted to 2% glucose-containing medium with DMSO and nocodazole for 2 additional hours, synchronized in G1 in 2% glucose-containing medium with α-factor for 2 h, and released into fresh 2% glucose-containing medium with 50 µg/ml pronase for 1 h.

### Flow cytometry

DNA content analysis was performed by flow cytometry as reported previously^36^. Cells were fixed with 70% ethanol, washed with phosphate-buffered saline (PBS 1X), incubated with 1 mg of RNaseA/ml PBS, and stained with 5 µg/ml propidium iodide. Samples were sonicated to separate single cells and analysed in a FACSCalibur flow cytometer.

### Plasmid supercoiling analysis

The distribution of topoisomers of the 2µ plasmid was performed as previously described^36^. Briefly, total DNA was extracted using a zymolyase-SDS standard protocol and run into 0.8% TPE 1 × agarose gels containing 4 μg/ml chloroquine for 36 h at 1.6 V/cm with recircularization. Negatively supercoiled topoisomers are resolved at this chloroquine concentration. Gels were blotted onto Hybond™-XL membranes and hybridized with a ^32^P-labeled *FLP1* fragment amplified by PCR from genomic DNA with oligos 5′-tgattacacataacggaaca-3′ and 5′-ttcagcactaccctttagc-3′. Signals were acquired in a Fuji FLA5100 and quantified with the ImageGauge analysis program. The total DNA signal (area under the curve) of the raw topoisomer profiles was equalized to eliminate DNA loading differences.

### Chromatin analysis by MNaseI digestion and indirect-end labeling

Chromatin analyses by MNaseI and indirect-end labeling were performed as previously described^57^. Briefly, cells were fixed for 15 min with 1% formaldehyde. Glycine was added to quench the reaction at a final concentration of 125 mM. Cells were sedimented, washed twice with cold PBS and stored at − 80 °C until use. Extracts for MNase digestion were resuspended in 1 M sorbitol/50 mM Tris HCl and digested for 1 h at 30 °C with 4.5 mg of zymoliase 20 T (AmsBio 120491-1) in gentle shaking. Samples were washed first with cold solution I (1 M sorbitol, 20 mM Tris HCl, 1 mM EDTA, 150 mM NaCl) and then with cold solution I plus 0.1 mM PMSF, then resuspended gently in cold solution II (20 mM Tris HCl, 1 mM EDTA, 150 mM NaCl, 0.1 mM PMSF, 0.2% Triton), and finally treated for 20 min at 37 °C with different concentrations of MNase (SIGMA N3755). The reaction was then stopped by adding 3 mM EDTA/4 mM Tris HCl and 10% SDS. To revert crosslinking, samples were incubated for 90 min at 37 °C with 1.5 mg of proteinase K and then overnight at 65 °C. DNA was extracted from samples using a standard phenol–chloroform extraction, treated with RNase A and loaded in a 1% agarose gel to check MNase digestion. MNase digestions used for indirect end labelling were incubated with *Eco*RI, resolved in 1.5% agarose gels, blotted onto a HybondTM-XL membrane and probed with a ^32^P-labeled specific PCR fragment located close to one of the *Eco*RI sites (oligos 5′- ataccaattcctcttcctag-3′ and 5′-tccaaatatacaagtggatc-3′). Signals were acquired in a Fuji FLA5100 with the Image Gauge analysis program.

### Chromatin analysis by MNase-seq

Chromatin analyses by MNase-seq were performed as previously described^57^. Briefly, MNaseI–digested DNA samples from two (G1 and G2) or one (S phase) biological replicates for each yeast strain were obtained as previously indicated for indirect-end labelling. MNase digested samples enriched in mononucleosomes were loaded in a 1% agarose gel, and the DNA corresponding to mononucleosomes was purified with a DNA purification kit (Bioline; BIO-52059). The DNA size and quality was confirmed by an electropherogram analysis (2100 Bioanalyzer™). Library construction and sequencing was performed at Genomics Core Facility of CABIMER. DNA libraries were prepared from 10 ng mononucleosome DNA using the TruSeq Chip Library Preparation kit (Illumina), and the size distribution and molarity of each library were analyzed with the Agilent™ DNA High Sensitivity Kit (Agilent 2100 Bioanalyzer). DNA libraries were sequenced on the NextSeq 500 Sequencing System (Illumina), and raw data were processed for basecalling, filtering and trimming to generate the FASTQ files using the BaseSpace Onsite v3.22.91.158 Software de Illumina. Sequence reads were mapped to *S. cerevisiae* genome sacCer3 by BowTie2^71^, and potential PCR duplicates were removed by SAM Tools on the Galaxy platform (usegalaxy.org)^72^. The peak-calling algorithm *Dpos* function (DANPOS 2.2.0)^48,73^ was used for nucleosome occupancy maps and comparative analyses using default parameters. Average nucleosome occupancy patterns flanking transcription start sites (TSS) from one (Fig. S2A) or two (Fig. 2) biological replicates were plotted in average density maps using *Profiles* function (DANPOS 2.2.0)^48,73^.

### Genome-wide data

Nucleosome profiles along the genome were visualized using the Integrative Genome Viewer (IGV)^74^.

### Western blot

Yeast protein extracts were prepared using the TCA protocol^75^ and resolved on a 8% SDS-PAGE. Rad53 was detected by standard western blot analysis with the rabbit polyclonal antibody JDI48^76^.

## Supplementary Information

Supplementary Information.

## Data Availability

The data that supports the findings of this study are available from the corresponding author upon reasonable request. Unique biological materials used in this study are available from the corresponding author. Raw data from MNase-seq have been deposited at the MIAME-compliant Gene Expression Omnibus (GEO) database at the National Center for Biotechnology Information (http://www.ncbi.nlm.nih.gov/geo/), and are accessible through the accession number GSE228861.
