# Supplementary material for: Transcription and FACT facilitate the restoration of replication-coupled chromatin assembly defects
Source: Sci Rep. 2023 Jul 14;13:11397. doi: 10.1038/s41598-023-38280-w (PMC10349138; doi:10.1038/s41598-023-38280-w)
Supplement: Supplementary file 1 — Supplementary Information. [file 41598_2023_38280_MOESM1_ESM.pdf]

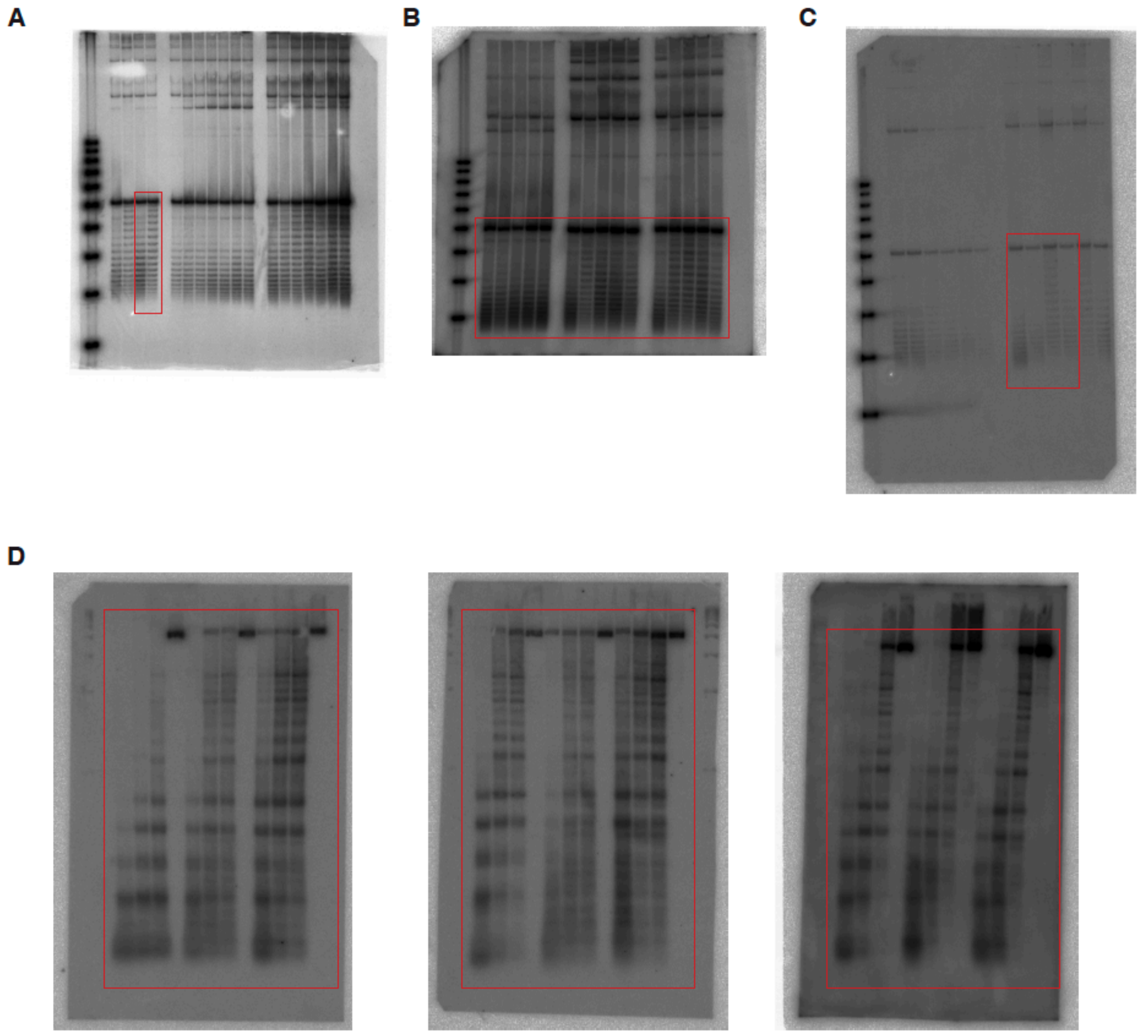

Figure S1. Original gels for Figure 1

Complete and cropped images for panels in Figures 1A-D.

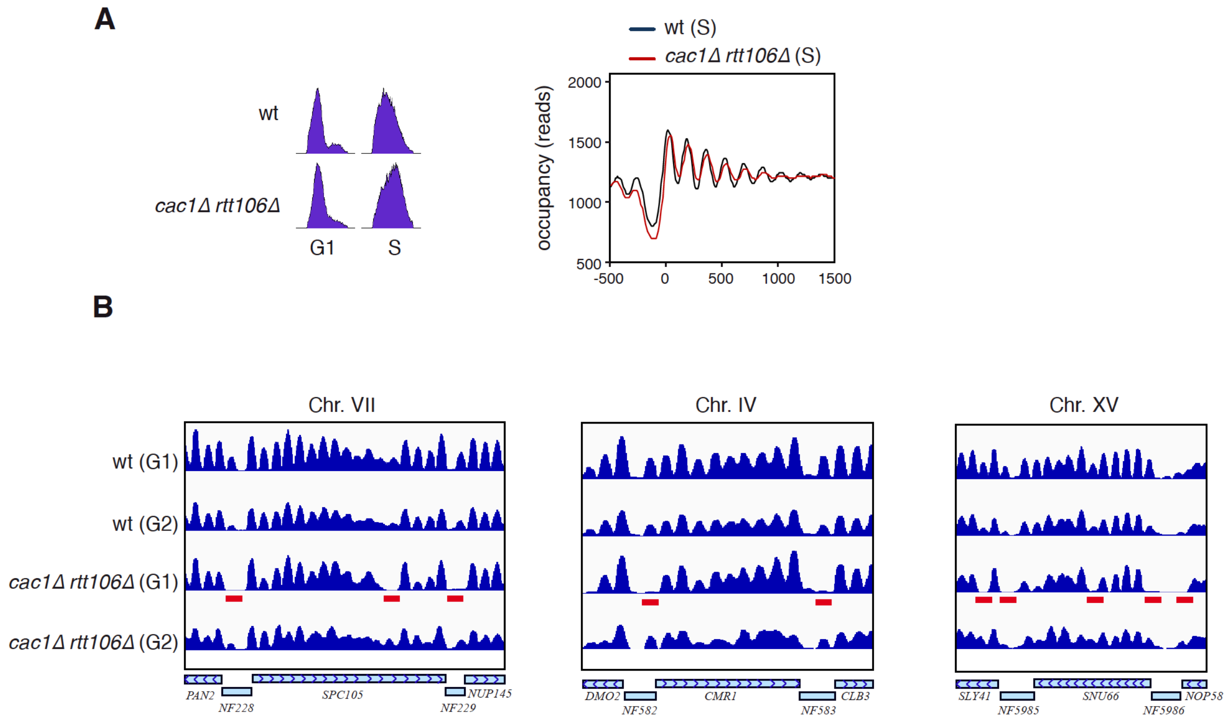

Figure S2. Genome-wide analysis of chromatin in *cac1*Δ *rtt106*Δ and wild type cells

(A) Genome-wide nucleosome profiles by MNase-seq for all yeast genes aligned relative to the TSS of wild type and *cac1*Δ *rtt106*Δ cells synchronized in G1 and released into fresh medium for 30 minutes until S phase. The FACS profiles are shown on the left. The analysis was performed with one biological replicate.

(B) Representative nucleosome profiles of wild type and *cac1*Δ *rtt106*Δ cells synchronized in G1 and released into fresh medium for 60 minutes until G2. Lost nucleosomes in *cac1*Δ *rtt106*Δ cells in G1 are underlined in red.

A

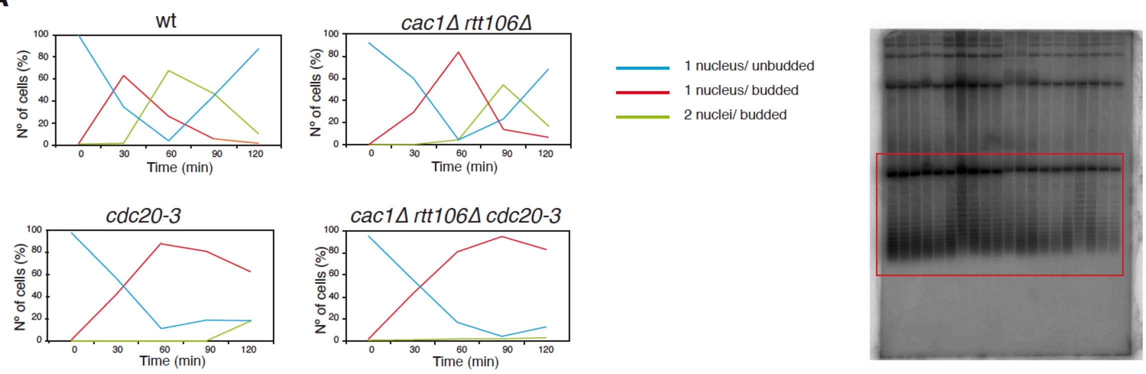

wt
cac1Δ rtt106Δ
cdc20-3
cac1Δ rtt106Δ cdc20-3
Nº of cells (%)
Time (min)
1 nucleus/ unbudded
1 nucleus/ budded
2 nuclei/ budded

B

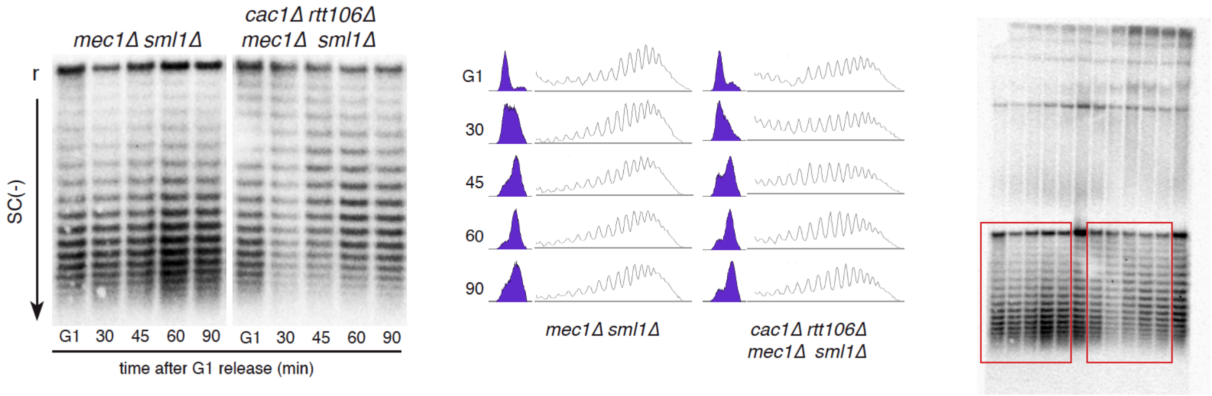

mec1Δ sml1Δ
cac1Δ rtt106Δ
mec1Δ sml1Δ
r
SC(-)
G1 30 45 60 90 G1 30 45 60 90
time after G1 release (min)
G1
30
45
60
90
mec1Δ sml1Δ
cac1Δ rtt106Δ
mec1Δ sml1Δ

C

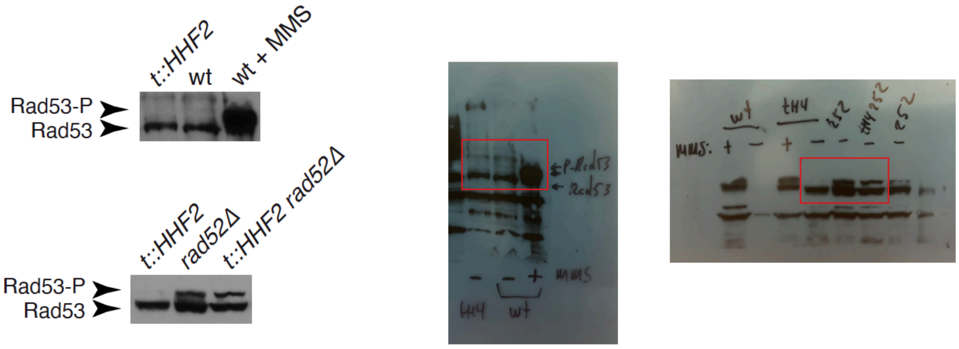

t::HHF2
wt
wt + MMS
Rad53-P
Rad53
t::HHF2
rad52Δ
t::HHF2 rad52Δ
Rad53-P
Rad53

E

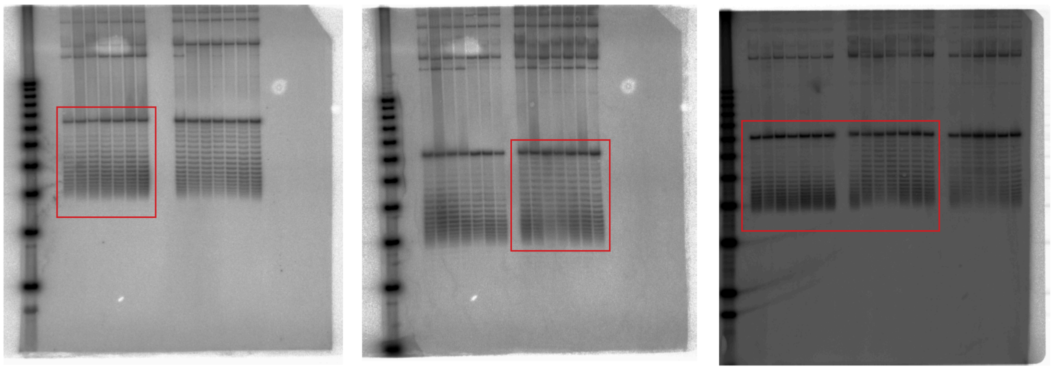

Figure S3. Controls for the analysis of the role of metaphase arrest on chromatin restoration

(A) Cell cycle progression of wild type, *cac1*Δ *rtt106*Δ, *cdc20-3*, and *cac1*Δ *rtt106*Δ *cdc20-3* cells under the conditions described in Figure 3A, as determined by cell morphology and DAPI staining of nuclei. The original gel for the topoisomer kinetics is shown on the right.

(B) Plasmid topoisomer distribution of the 2µ plasmid in *mec1*Δ *sml1*Δ and *cac1*Δ *rtt106*Δ *mec1*Δ *sml1*Δ cells synchronized in G1 and released into fresh medium for different times. Cell cycle progression and topoisomer profiles are shown on the right.

(C) Partial depletion of histone H4 does not activate the DDC. DDC as determined by phosphorylation of Rad53 in asynchronous cultures of wild type and *t::HHF2* cells. Wild type cells treated with 0.033% MMS were included as positive control (top panel). The lack of SAC activation in *t::HHF2* cells is not due to active homologous recombination, as *rad52*Δ and *t::HHF2 rad52*Δ cells display similar levels of Rad53 phosphorylation (bottom panel). Original gels and selected areas are shown on the right.

(D) Original gels for Figure 3B. The selected areas for the corresponding panels are marked in red.

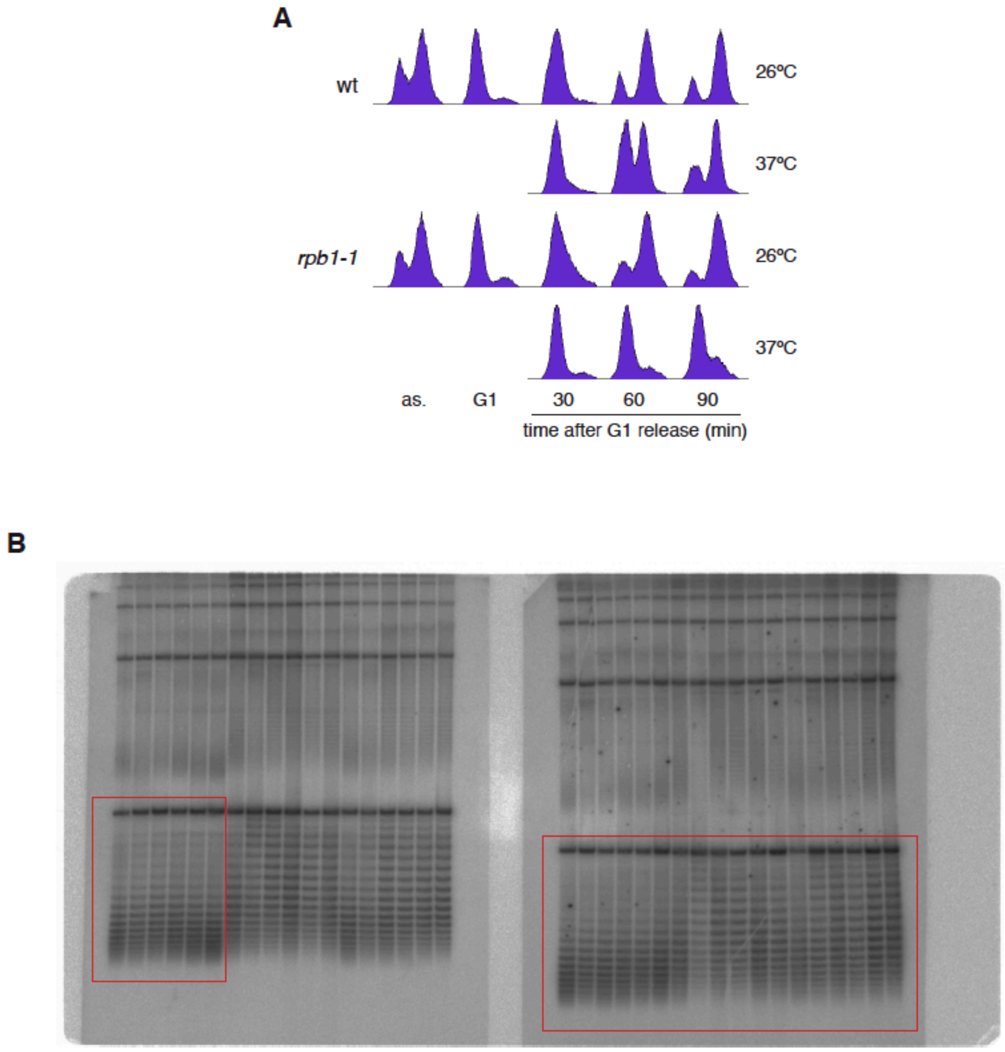

Figure S4. The *rpb1-1* mutant is defective in G1 release at restrictive temperature

(A) The *rpb1-1* mutant is defective in G1 release at restrictive temperature. Cell cycle progression of wild type and *rpb1-1* cells synchronized in G1 and released into fresh medium for different times at 26°C or 37°C.

(B) Original gels for Figure 4. The selected areas for the corresponding panels are marked in red.

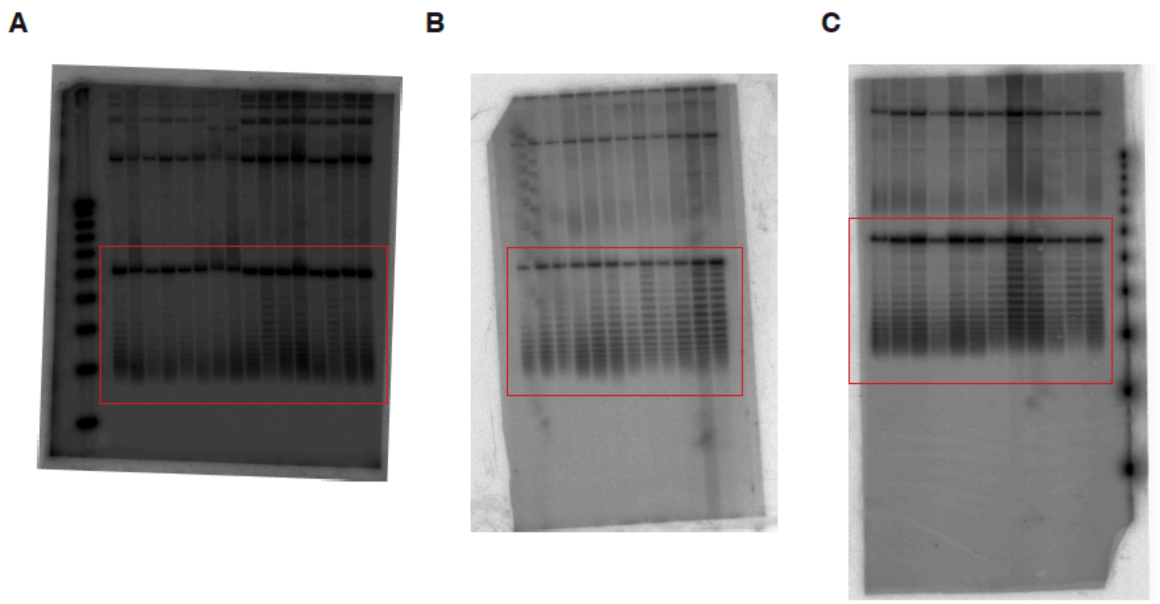

Figure S5. Original gels for Figure 5

The selected areas for the corresponding panels are marked in red.

Table S1. ***Saccharomyces cerevisiae*** **strains used in this study**

**Strain**	***Genotype***	**Ref.**	**Fig.**
w303b (YK-402)	*MATa leu2-3,112 trp1-1 ura3-1 ade2-1 can1-100 his3-11 RAD5bar1Δ::HISG*	[1]	1A, 1B, 1D S1A, S1B, S1D, 2, S2B, 3, S3A, S3E, 5, S5
wtH4-9b	*bar1Δ::natMX4 hhf1Δ::hygMX4 hhf2Δ::kanMX4 (p413TARtetH4)*	[2]	1A, S1A, 1D, S1D, 3B, S3E
wtH4-8d	*bar1Δ::natMX4 hhf1Δ::hygMX4 hhf2Δ::kanMX4 (p413TARtetH4)*	[3]	1B, S1B
DMY10-10b	*bar1Δ::natMX4 cac1Δ::kanMX4 rtt106Δ::hygMX4 hmlΔ::LEU2*	This work	1B, 1D, S1B, S1D, 2, S2B, 3A, S3A, 5, S5
DMY42-14a	*bar1Δ::LEU2 cdc6:TRP1:GAL-CDC6*	This work	1C, S1C
DMY37-7c	*bar1Δ::LEU2 hhf1Δ::hygMX4 hhf2Δ::kanMX4 (p413TARtetH4) cdc6:TRP1:GAL-CDC6*	This work	1C, S1C
DMY87.1-3b	*bar1Δ::natMX4 cdc20-3*	This work	3A, S3A
DMY90a-21d	*bar1Δ::natMX4 cac1Δ::kanMX4 rtt106Δ::hygMX4 hmlΔ::LEU2 cdc20-3*	This work	3A, S3A
wm2-2	*bar1Δ::hisG mad2Δ::LEU2*	[2]	3B, S3E
wtH4bm2-1	*bar1Δ::natMX4 hhf1Δ::hygMX4 hhf2Δ::kanMX4 (p413TARtetH4) mad2Δ::LEU2*	[2]	3B, S3E
wmec1s1-2c	*bar1Δ::natMX4 mec1Δ::LEU2 sml1Δ::URA3 hmlΔ::LEU2*	This work	S3B
wc1r106m1s1-21d	*bar1Δ::natMX4 cac1Δ::kanMX4 rtt106Δ::hygMX4 mec1Δ::LEU2 sml1Δ::URA3 hmlΔ::LEU2*	This work	S3B
w303-11d	*bar1::LEU2*	This work	S2A, 4, S4
wrpb1-1-3a	*bar1::LEU2 rpb1-1*	This work	4, S4

wc1r106-7c	*bar1Δ::natMX4 cac1Δ::kanMX4 rtt106Δ::hygMX4 hmlΔ::LEU2*	This work	S2A, 4, S4B
wc1r106r1-1_3	*cac1Δ::kanMX4 rtt106Δ::hygMX4 hmlΔ::LEU2 bar1Δ::natMX4 rpb1-1*	This work	4, S4B
DMY84.1	*bar1Δ::HISG hir1Δ::HIS3*	This work	5A, S5A
DMY86.1	*bar1Δ::natMX4 cac1Δ::kanMX4 rtt106Δ::hygMX4 hmlΔ::LEU2 hir1Δ::HIS3*	This work	5A, S5A
DMY161-5c	*bar1Δ::natMX4 spt16G132D*	This work	5B, S5B
DMY165-3a	*bar1Δ::natMX4 cac1Δ::kanMX4 rtt106Δ::hygMX4 hmlΔ::LEU2 spt16G132D*	This work	5B, S5B
DMY155-39c	*bar1Δ::natMX4 hmlΔ::LEU2 spt16-m::natR*	This work	5C, S5C
DMY151-32a	*bar1Δ::natMX4 cac1Δ::kanMX4 rtt106Δ::hygMX4 hmlΔ::LEU2 spt16-m::natR*	This work	5C, S5C
BY4741	*Mat a his3Δ1 leu2Δ0 ura3Δ0 met15Δ0 LYS2*	Euroscarf	S3C
BYtetH4-10D	*Mat α MET15 LYS2 hhf1Δ::kanMX4 hhf2Δ::kanMX4 (p413TARtetH4)*	[4]	S3C
Y10540	*Mat α MET15 lys2Δ0 rad52Δ::kanMX4*	Euroscarf	S3C
BY52tetH4-2D	*Mat α MET15 LYS2 hhf1Δ::kanMX4 hhf2Δ::kanMX4 (p413TARtetH4) rad52Δ::kanMX4 trp1Δ::kanMX4*	[4]	S3C

All strains are isogenic to W303-1A (*MATa leu2-3,112 trp1-1 ura3-1 ade2-1 can1-100 his3-11 RAD5*), except BY and Y1 strains that are isogenic to BY4741 (*Mat a his3Δ1 leu2Δ0 ura3Δ0 met15Δ0 LYS2*). Only the relevant genotypes are shown.

References

1. Ogi, H., Wang, C.-Z., Nakai, W., Kawasaki, Y. & Masumoto, H. The role of the Saccharomyces cerevisiae Cdc7–Dbf4 complex in the replication checkpoint. *Gene* 414, 32–40 (2008).

2. Murillo-Pineda, M., Cabello-Lobato, M. J., Clemente-Ruiz, M., Monje-Casas, F. & Prado, F. Defective histone supply causes condensin-dependent chromatin alterations, SAC activation and chromosome decatenation impairment. *Nucleic Acids Res* 42, 12469–12482 (2014).

3. Maya-Miles, D. *et al.* Crosstalk between chromatin structure, cohesin activity and transcription. *Epigenet Chromatin* 12, 47 (2019).

4. Prado, F. & Aguilera, A. Partial Depletion of Histone H4 Increases Homologous Recombination-Mediated Genetic Instability. *Mol Cell Biol* 25, 1526–1536 (2005).
